# Degradation-Grafting Mechanochemical Treatment of Recycled Polypropylene as an Asphalt Modifier: Rheological Behavior and Microscopic Mechanisms

**DOI:** 10.3390/polym18202545

**Published:** 2026-10-16

**Authors:** Jun Zhong, Xiong Xu, Sha Wu, Hui Tong, Shuang Zhou, Jie Liu, Song Xu, Zongwu Chen

**Affiliations:** 1 Hubei United Transportation Industrial Development Co., Ltd., Wuhan 430056, China; zhongjun86@126.com (J.Z.); 18120401039@163.com (S.W.); 15377077647@163.com (H.T.); 15623873256@163.com (S.Z.); 2 Wuhan Metropolitan Area Investment and Development Group Co., Ltd., Hubei Communications Investment Group Co., Ltd., Wuhan 430040, China; 3 School of Civil Engineering and Architecture, Wuhan Institute of Technology, Wuhan 430074, China; 21090101@wit.edu.cn; 4 Hubei Provincial Engineering Research Center for Green Civil Engineering Materials and Structures, Wuhan Institute of Technology, Wuhan 430074, China; 5 College of Civil Engineering, Fuzhou University, Fuzhou 350108, China; xusong@fzu.edu.cn; 6 School of Materials Science and Engineering, Chang’an University, Xi’an 710061, China; chenzw@cug.edu.cn

**Keywords:** recycled polypropylene (RPP), modified asphalt, reaction mechanism, rheological properties

## Abstract

As a common solid waste, recycled polypropylene (RPP) has been demonstrated to be a viable asphalt modifier for enhancing pavement performance. However, its direct application still faces bottlenecks including high processing temperatures, poor dispersion, and unstable performance, which hinder its potential promotion. To address this, this study adopted a mechanochemical treatment of RPP with varying dosages of di-cumyl peroxide (DCP) and epoxidized soybean oil (ESO) to prepare a novel waste plastic asphalt modifier. This process was designed to cleave the long molecular chains of RPP via DCP, while simultaneously allowing dynamic chemical grafting reactions with ESO, yielding a degraded WPP bearing flexible functional groups. Through rheological temperature and frequency sweeps, Fourier transform infrared spectroscopy (FTIR), and mechanistic analysis, the intrinsic correlation between the microstructure and macroscopic rheological behavior of the modified binder was systematically investigated. Among the investigated formulations, the results indicated that 0.3RPP-MA showed the highest viscoelastic transition temperature, 25.7 °C, compared with 7.8 °C for the virgin binder. The dynamic shear rheology (DSR) results indicate that this formulation provided the most favorable high-temperature rheological response within the tested DCP range. FTIR spectra clearly verified the formation of ESO graft copolymers and the functional group changes induced by DCP degradation, and a reaction mechanism involving simultaneous degradation and grafting was elucidated. Overall, the preparation method proposed in this study provides a comprehensive analysis of the rheological properties of RPP-modified asphalt, providing both engineering guidance and theoretical support for the high-value utilization of waste plastics.

## 1. Introduction

The continuous expansion of road infrastructure worldwide has led to an unprecedented demand for paving materials [1,2,3]. However, conventional petroleum asphalt pavements are increasingly struggling to meet performance requirements under extreme climatic conditions, such as severe temperature fluctuations, and high load traffic, both of which frequently accelerate pavement distress [4,5]. Meanwhile, the massive accumulation of polypropylene (PP) plastic waste has become an urgent environmental concern on a global scale, as PP refuse not only occupies substantial landfill space but also poses long-term ecological risks due to its resistance to natural degradation [6,7]. Moreover, current disposal approaches, predominantly incineration and landfilling, are not only of questionable environmental sustainability but also fail to generate added value [8,9]. In this context, the present study aims to explore the use of recycled polypropylene (RPP) as an asphalt modifier, thereby providing a feasible strategy to simultaneously enhance pavement service performance and alleviate plastic pollution.

Considerable research efforts have been devoted to modifying asphalt with waste polymer materials [10,11]. The unique chemical structure of plastics, characterized by long-chain saturated hydrocarbon backbones, endows them with excellent thermal stability and outstanding resistance to permanent deformation at elevated temperatures, making them inherently suitable for enhancing the high-temperature performance of asphalt pavements [12,13,14]. To date, three mainstream processing methods have been established for incorporating waste plastics into asphalt systems [15]. Brovelli et al. [16] employed the wet process, in which waste plastics are melted and directly blended into hot asphalt binder prior to mixing with aggregates. This method has been widely adopted for its ability to significantly improve the rutting resistance, extend the fatigue life, and enhance the overall durability of the modified binder. In contrast, Miranda et al. [17] adopted the dry process, which introduces waste plastics in solid form directly during the aggregate mixing stage. This method does not require pre-melting or specialized mixing equipment, offering notable advantages in terms of operational simplicity, reduced energy consumption, and lower production costs.

Although both the wet and dry processes have demonstrated technical feasibility, the direct incorporation of RPP into asphalt binder remains severely constrained by inherent drawbacks [13,18,19], such as its relatively high melting temperature and poor compatibility with the asphalt phase [20,21]. The melting point of RPP typically exceeds 180 °C, requiring processing temperatures far above the conventional asphalt mixing range [22,23]. Such elevated temperatures not only incur a significant energy penalty but also accelerate emissions of volatile organic compounds and other harmful by products [24,25,26], while the high temperature simultaneously promotes thermal oxidative aging of the asphalt binder, thereby impairing its long-term service performance [13,27,28]. On the other hand, it can lead to severe phase separation during storage, transportation, and application between the marked polarity difference and molecular weight disparity between RPP and the aromatic and asphaltene fractions in asphalt [17,29]. All these factors severely limit the practical application feasibility of RPP-modified asphalt.

The pre-treatment of RPP prior to asphalt modification has recently emerged as a more efficient approach [30,31]. Javadi et al. [32] increased the modification temperature and prepared PP-modified asphalt at 180 °C. Dynamic shear rheometer (DSR) tests revealed a significant improvement in high temperature performance, and fluorescence microscopy (FM) confirmed a relatively uniform dispersion of the modifier within the asphalt. Dalhat and Al-Abdul Wahhab [33] used ground PP and PE plastics to prepare waste-plastic-modified asphalt. In their study, multiple-stress creep recovery (MSCR) tests demonstrated that this method enhanced the rutting resistance compared with untreated plastics. Li et al. [34] pyrolyzed PP and used the resultant waxy materials to produce modified asphalt, finding that the modifier could be melted at a lower temperature and that the viscosity of the modified asphalt was markedly reduced. However, the rheological properties of the modified asphalt under various conditions have not been comprehensively investigated, and the specific mechanisms underlying the performance enhancement via thermochemical degradation and functional group grafting remain unclear.

**Figure 1:**
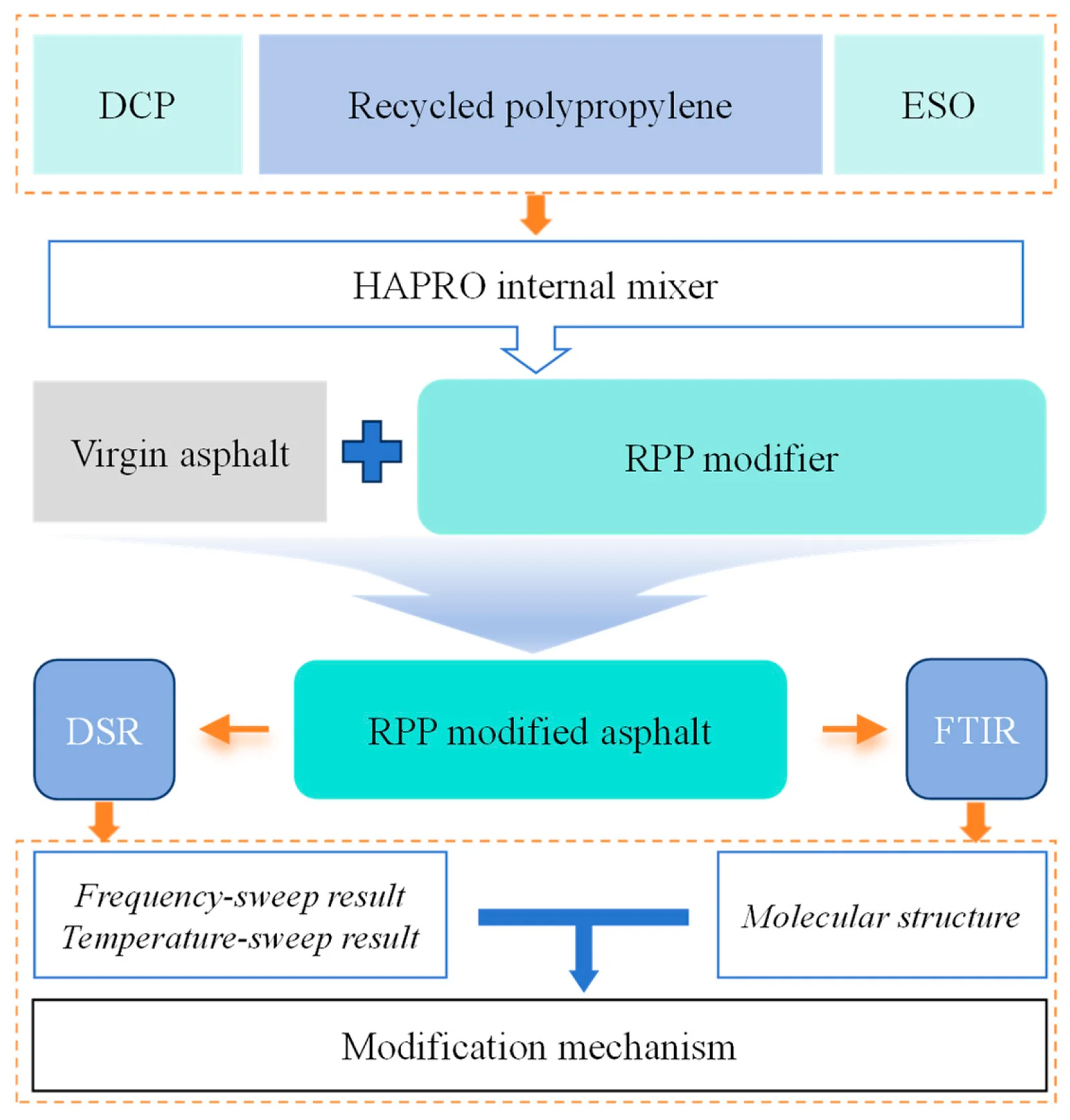
Flowchart of this research plan.

To fill the research gap, this study treats RPP with different formulations of DCP and ESO. The free radicals generated by thermal decomposition not only enable the degradation of PP but also promote the ring-opening of the epoxy groups in ESO and their grafting onto RPP. This treatment not only introduces flexible groups into the rigid PP backbone but also lowers the melting temperature of the polymer, thereby optimizing the performance of the RPP-modified asphalt. On this basis, temperature sweep tests were conducted on the asphalt using DSR, followed by frequency sweep analyses at various temperatures after fitting with the CA model, to systematically evaluate its physical properties. In addition, FTIR spectroscopy was employed to analyze the changes in chemical functional groups before and after the reaction, thereby elucidating the modification mechanism. Finally, this study is expected to provide a clean pathway for the high-quality recycling of RPP into pavement materials. The flowchart of the experimental scheme is presented in Figure 1.

## 2. Materials and Methodology

### 2.1. Raw Materials

#### 2.1.1. Virgin Asphalt Binder

The virgin asphalt binder used in this experiment was 70# petroleum asphalt, supplied by Lanoujiancai Co., Ltd. in Wuhan City, China. The asphalt was stored in a cool, dry indoor environment, with the storage temperature controlled below ambient conditions (approximately 25 °C) to minimize thermal oxidative aging and the volatilization of light components. Prior to each test, the asphalt containers were placed in an oven and heated at 160 °C until completely molten and fluid, ensuring uniform temperature throughout the binder and the absence of entrained air bubbles. According to the relevant stardant ASTM D36, ASTM D4402, ASTM D113 and ASTM D5 [35,36,37,38] the measured values of its key physical performance indicators and primary rheological parameters are uniformly summarized in Table 1.

#### 2.1.2. RPP

**Figure 2:**
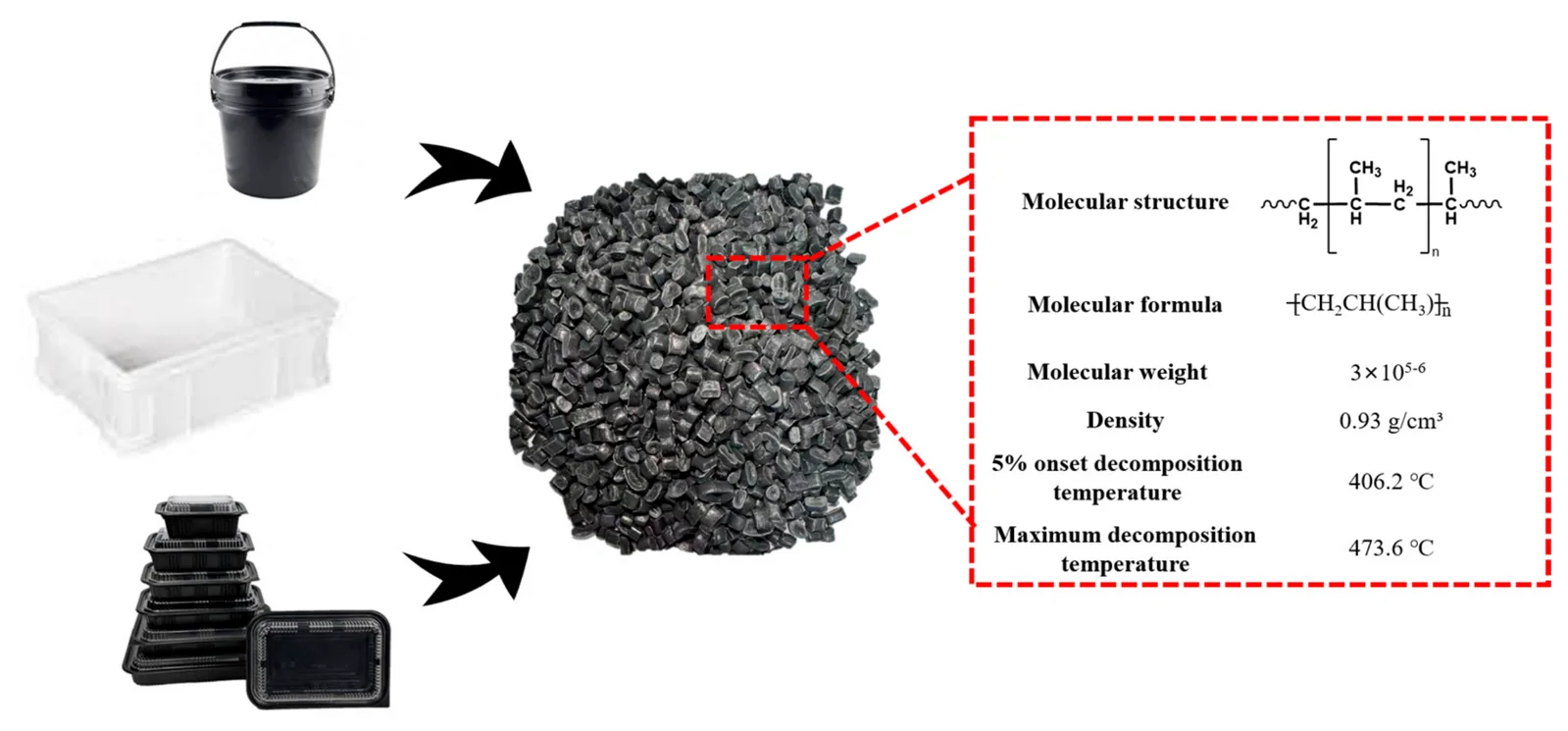
Physical and chemical properties of RPP.

The PP pellets used in this study were obtained from discarded plastic packaging materials, primarily food packaging containers, daily-use plastic products, and some industrial turnover boxes, collected from household waste sorting in Wuhan and its surrounding areas. They were supplied by a local recycling enterprise qualified for the resource utilization of waste plastics. Figure 2 shows recycled pellets, approximately 3–5 mm in diameter and cylindrical in shape, which were produced through a series of processes including manual sorting, crushing, hot alkaline water washing, dewatering, melt extrusion, water-cooled pelletizing, and homogenization. The pellets exhibited a color ranging from light gray to grayish black, with a slight unevenness in hue. Minor dark spots were observable on the pellet surfaces, but no visible hard impurities such as fiber, metals, or sand grains were detected. Prior to testing, the required amount of recycled PP pellets was dried in an oven at 80 °C for 4 h to remove surface adsorbed moisture, and then cooled to room temperature before use.

#### 2.1.3. Dicumyl Peroxide (DCP)

**Figure 3:**
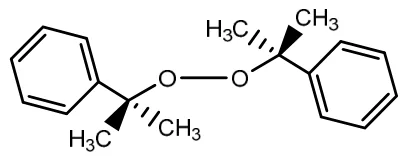
Molecular structure of used DCP [13].

The chemical degradation agent used in this study was dicumyl peroxide (DCP)It was purchased from Shanghai Macklin Biochemical Co., Ltd. (Shanghai, China). It is a white crystalline powder, insoluble in water but soluble in organic solvents such as benzene, cumene, diethyl ether, and petroleum ether. The reagent was stored in a dedicated explosion-proof refrigerated cabinet at a controlled temperature of 2–8 °C and was strictly protected from light. In this work, DCP served as both a degradation agent and a free-radical initiator, with the aim of generating free radicals through thermal decomposition to induce chain-scission degradation reactions among polymer chains or to exert a reactive compatibilization effect. The molecular structure and key physicochemical parameters of DCP are summarized in Figure 3 and Table 2.

#### 2.1.4. Epoxidized Soybean Oil (ESO)

**Figure 4:**
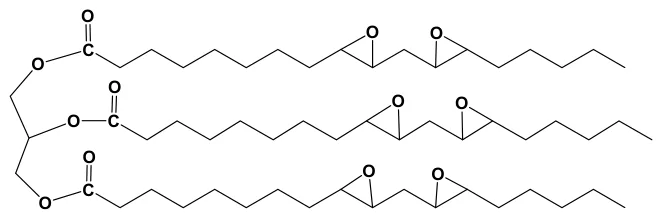
Molecular structure of ESO used in the study [13].

The epoxidized soybean oil (ESO) used in this study was purchased from Shanghai Macklin Biochemical Co., Ltd. (Shanghai, China). The product was produced from refined soybean oil via an in situ epoxidation method using performic acid, without the addition of any extra diluents or antioxidants. At ambient temperature, ESO appears as a transparent oily liquid ranging from light yellow to pale amber, with moderate viscosity (fluid at 25 °C), and is free from visible suspended matter or precipitation. The reagent exhibits good hydrophobicity and is insoluble in water. In this work, ESO serves a dual role as both a plasticizer and a reactive compatibilizer through its epoxy functional groups, which can interact synergistically with the polar components in asphalt or with the reactive radical sites generated from DCP decomposition. The molecular structure and key physicochemical properties of ESO are summarized in Figure 4 and Table 3.

### 2.2. Preparation of RPP Modifier and RPP-Modified Asphalt

Prior to the preparation of the RPP asphalt modifier, the RPP pellets were placed in an oven and dried at 80 °C for 4 h until no moisture remained. RPP, DCP, and ESO were weighed to 40 g, thoroughly mixed, and then charged in a HAPRO internal mixer (Harbin Hapro Electrical Technology Co. Ltd, Wuhan City, China). The mixer was set to an internal temperature of 180 °C and operated for 10 min. The resulting modifier was allowed to cool to room temperature, then crushed and sieved through a 30–mesh screen to obtain the desired RPP asphalt modifier. The RPP: DCP: ESO reagent ratios were 100:0:5, 100:0.3:5, 100:0.6:5, and 100:0.9:5, and the modifiers were designated 0RPP, 0.3RPP, 0.6RPP, and 0.9RPP, accordingly.

After obtaining the different RPP asphalt modifiers, they were added to the molten asphalt at a concentration of 10% by weight of the asphalt. First, the mixture was stirred at 160 °C using a low-speed mixer for 40 min until the modifier was uniformly dispersed. Subsequently, it was sheared at 160 °C with a high-speed shear mixer at a rotation speed of 4000 rpm for 40 min. Finally, the obtained asphalt was placed in an oven at 160 °C and held for 1 h to allow complete dissolution of the modifier. The resulting RPP-modified asphalts, corresponding to the different modifier formulations, were named 0RPP-MA, 0.3RPP-MA, 0.6RPP-MA, and 0.9RPP-MA, respectively. The meanings represented by different abbreviations show the modified asphalt from waste plastic obtained by degrading RPP using 0, 0.3%, 0.6%, and 0.9% DCP.

### 2.3. Test Methodologies

#### 2.3.1. Temperature-Dependent DSR Test

**Figure 5:**
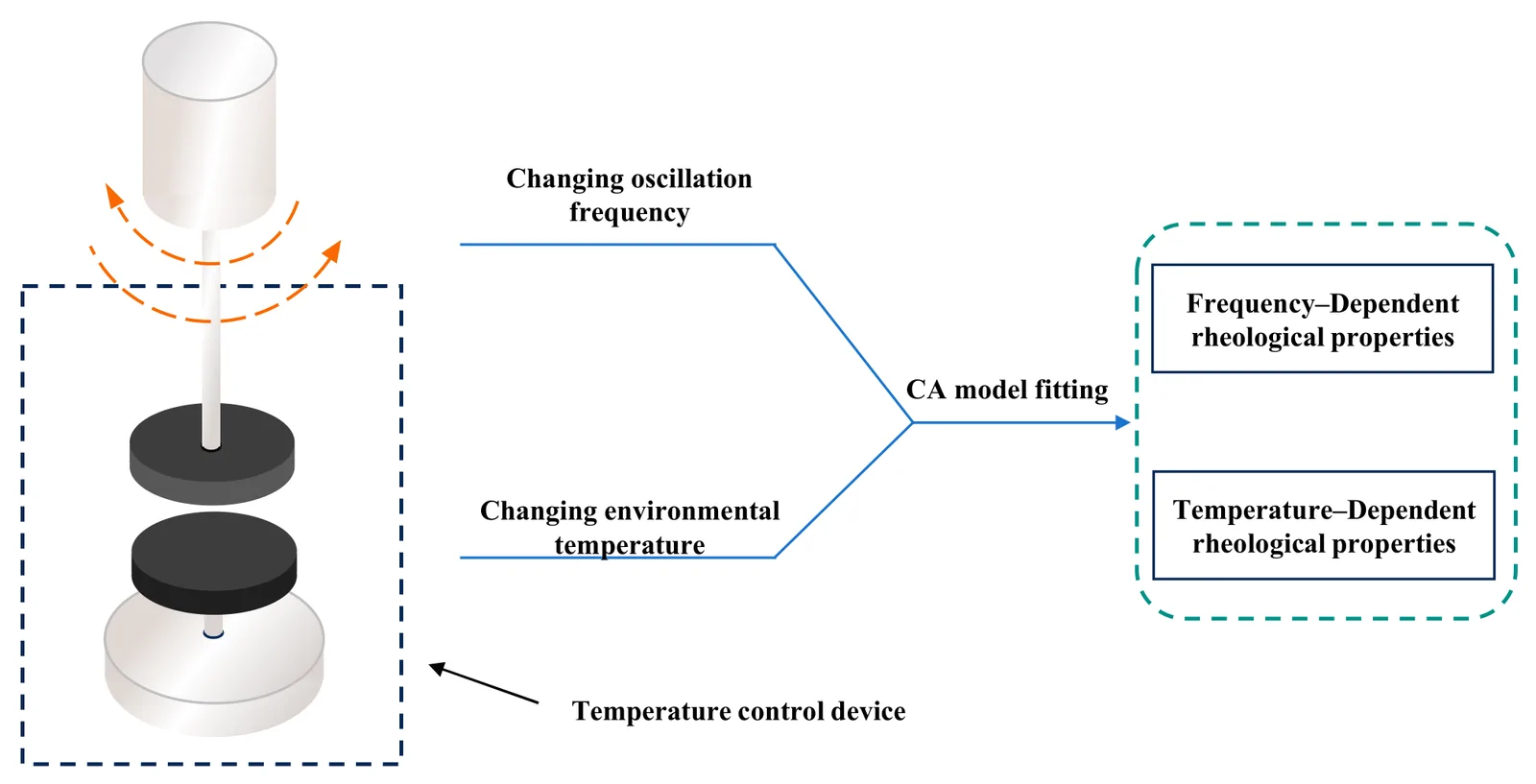
Main components of DSR testing.

In this study, temperature sweep tests were conducted using a DSR (SHANGHAI LREL INSTRUMENT CO.,LTD., Shanghai City, China) to systematically investigate the evolution of the viscoelastic properties of the asphalt binders with temperature. Under dynamic loading at various temperatures, the complex shear modulus (G*) and phase angle (δ) of the binders were measured, based on which the high-temperature rutting resistance, low-temperature cracking resistance, and temperature susceptibility of the RPP-modified asphalt before and after modification were evaluated [39]. To simulate actual field conditions, the tests were performed at a fixed frequency of 10 rad/s over a temperature sweep range from 4 °C to 76 °C, with a heating rate of 2 °C/min. A parallel plate geometry with a diameter of 8 mm was used for testing temperatures below 28 °C, while a 25 mm diameter plate was employed for temperatures above 28 °C. The gap between the plates was maintained at 1 mm throughout. The relevant information for the DSR test is shown in Figure 5.

#### 2.3.2. Frequency-Dependent DSR Test

The dynamic shear rheological test (DSR) is a method to evaluate the shear properties of materials by analyzing their viscoelastic behavior under varying frequencies, thereby assessing the high-temperature rutting resistance and low-temperature fatigue cracking resistance of asphalt. In this study, an MCR–302 dynamic shear rheometer was used to conduct frequency sweep tests on asphalt before and after rejuvenation, evaluating its viscoelastic dynamic properties under simulated varying vehicle load conditions. Testing conditions included strain-controlled mode at 1%, with temperature ranging from 4 °C to 76 °C and loading frequencies spanning 0.1–100 rad/s. Based on temperature sweep DSR data, the time-temperature superposition principle was applied to construct a master curve using the Christensen–Anderson (CA) model, enabling the description and prediction of the mechanical behavior of asphalt across diverse temperature and loading conditions [40]. The mathematical framework for the master curve construction is outlined as follows [41]:

(1) $$\log_{a}\left(T\right)=\frac{-\;C_{1}(T\;-\;T_{r})}{C_{2}+T\;-{\;T}_{r}}$$

(2) $$\omega_{r}=\omega *10^{\log_{a}\left(T\right)}$$

(3) $${G^{*}}_{\mathrm{model}}={\;G}_{g}\cdot {[1+{\;(\frac{\omega_{r}}{\omega_{c}})}^{\frac{\log2}{R}}]}^{-\;\frac{R}{\log2}}$$

(4) $$\delta_{\mathrm{model}}=\frac{90}{[1+{\left(\frac{\omega_{r}}{\omega_{c}}\right)}^{\frac{\log2}{R}}]}$$

where *T* is test temperature, °C; *T_r_* is reference temperature, °C; *C_1_* and *C_2_* are fitting parameters; *ω* is the test frequency and *ω_r_* is the reduced frequency, respectively; *G_g_* is glass modulus, assumed to be equal to 1 GPa (1 × 10^9^ Pa); *ω_c_* is crossover frequency; *R* is rheological indexis rheological index; and *Gg*, *ω_c_* and *R* are the fitting parameters of the CA model.

#### 2.3.3. Rutting Factor and Fatigue Factor Analysis

The high-temperature rutting resistance of asphalt is typically quantified by the rutting factor, defined as G*/sin δ, which is the ratio of the complex shear modulus to the sine of the phase angle. A higher value of this factor indicates a greater capability of the recycled asphalt to resist permanent deformation at elevated temperatures. Correspondingly, the fatigue factor, expressed as G*·sin δ, the product of the complex shear modulus and the sine of the phase angle, serves as the key indicator for evaluating the fatigue-cracking resistance of asphalt. A lower value of the fatigue factor signifies superior fatigue performance of the recycled asphalt under cyclic loading, implying that an accumulation of fatigue damage is less prone to occur.

#### 2.3.4. Viscoelastic Transition Temperature Analysis

The viscoelastic transition temperature of asphalt is defined as the critical temperature at which the material transforms from an elastic state to a viscous flow state during heating. This parameter directly reflects the temperature-dependent viscosity response of the recycled asphalt, which is characterized by a gradual evolution in its physical state from solid or high-elastic to viscous fluid as the temperature rises. In this study, the viscoelastic transition temperature serves as an important parameter for evaluating the temperature susceptibility and service applicability of the RPP-modified asphalt.

#### 2.3.5. FTIR Test

In this study, the molecular structural changes in asphalt induced by the RPP modifier were characterized using a Nicolet iS20 FTIR spectrometer (Thermo Fisher Scientific (China) Co., Ltd., Wuhan City, China). The transmission infrared spectra of the RPP-modified asphalts across different wavenumber regions were qualitatively analyzed to identify the characteristics of functional group variations in the asphalt binders modified with RPP prepared at different ratios. Analysis of the characteristic functional groups was used to elucidate the chemical nature of the reactions among RPP, DCP, and ESO. The spectral acquisition parameters were set as follows: wavenumber range 4000–700 cm^−1^, resolution 4 cm^−1^, and 32 scans per spectrum.

## 3. Results and Discussion

### 3.1. Temperature Response to Rheological Properties

#### 3.1.1. Complex Modulus and Phase Angle

**Figure 6:**
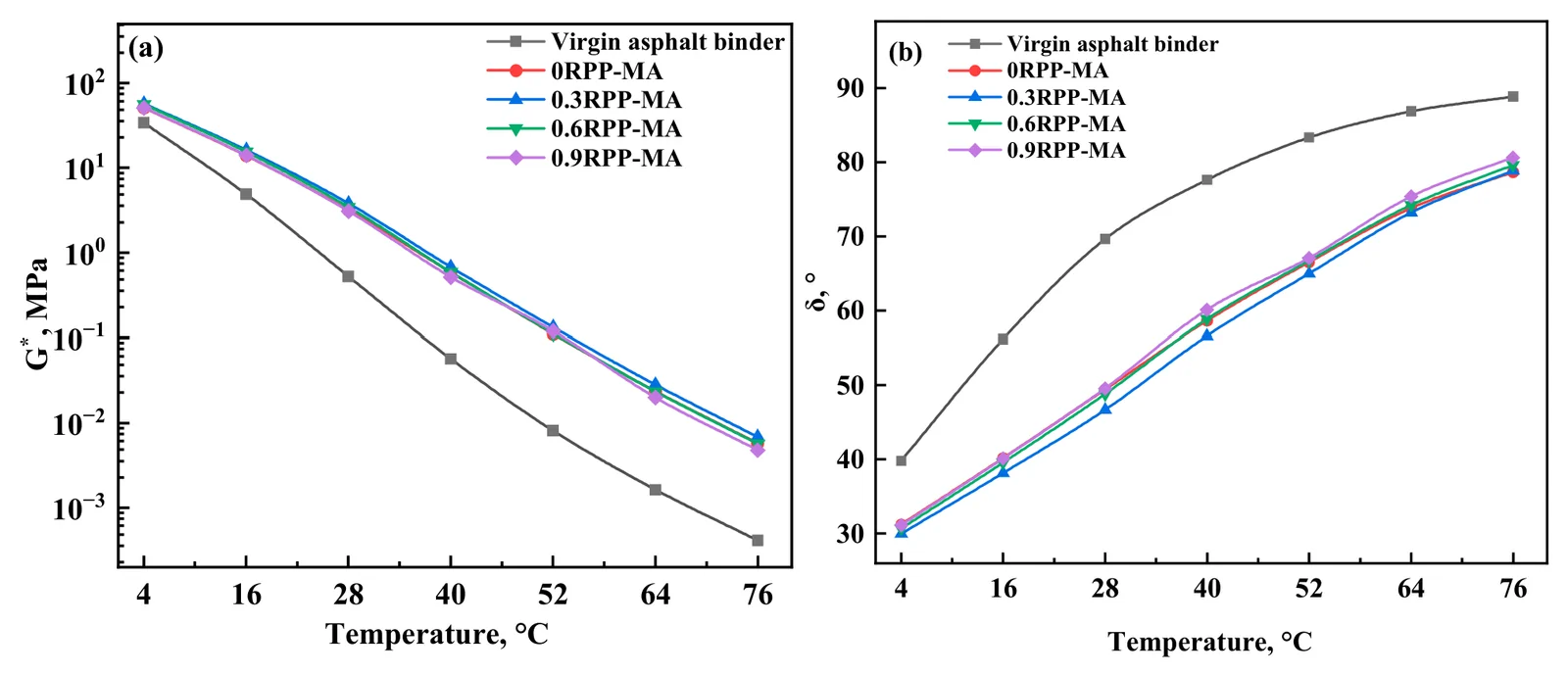
Complex modulus (**a**) and phase angle (**b**) at different temperatures for RPP-modified asphalt at 10 rad/s.

Figure 6 shows the measured complex shear modulus (G*) and phase angle (δ) of the asphalt at various temperatures under oscillatory loading at a frequency of 10 rad/s. It is evident that the G* values of the RPP-modified asphalt are consistently higher than those of the virgin asphalt, while the δ values are lower across the entire testing temperature range. This can indicate that the RPP modifier enhances the deformation resistance of the asphalt, and the modified binder exhibits a more pronounced elastic behavior in its viscoelastic response. This improvement can be attributed to the fact that PP is a high-molecular-weight polymer with greater stiffness than the virgin asphalt, particularly at elevated temperatures. Upon modification, the modifier is able to form a skeleton-like network structure within the asphalt matrix, thereby increasing the overall stiffness of the binder, as reflected by the increased complex shear modulus and the decreased phase angle.

#### 3.1.2. Rutting Factor and Fatigue Factor

**Figure 7:**
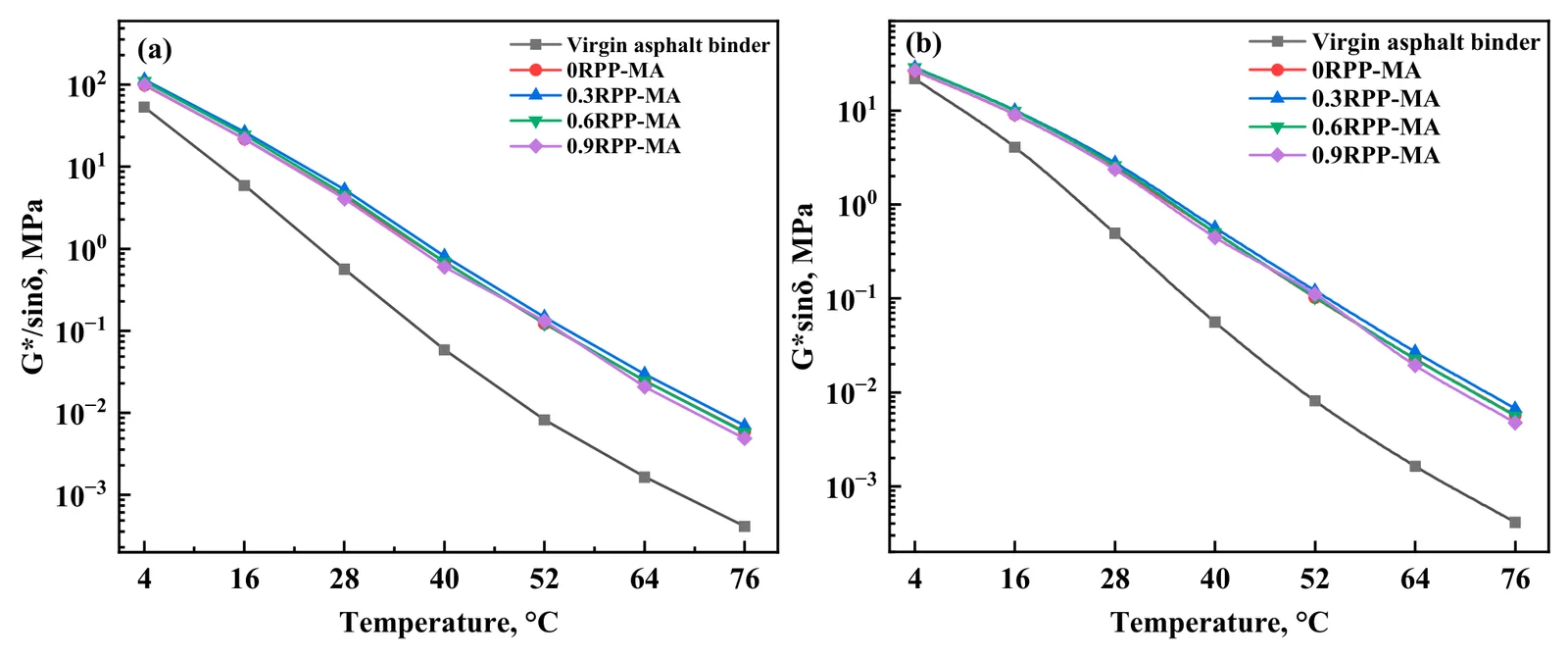
Rutting factor (**a**) and fatigue factor (**b**) at different temperatures for RPP-modified asphalt at 10 rad/s.

Figure 7a presents the rutting factor (G*/sin δ) results obtained from temperature sweep tests on the asphalt binders. It can be observed that the rutting factor values of the RPP-modified asphalts are consistently higher than those of the virgin asphalt across the entire testing temperature range. Among the RPP-modified binders, the one containing 0.3% DCP in the modifier formulation exhibits a slightly higher rutting factor compared to the others. These results can indicate that the incorporation of the RPP modifier improves the rutting resistance of the asphalt, particularly at elevated temperatures. Among all the tested RPP-modified asphalts, the 0.3RPP-MA formulation shows the most prominent performance. This enhancement is attributed to the increased stiffness and viscosity of the asphalt upon PP addition, which renders the modified binder more resistant to flow and deformation at high temperatures, thereby strengthening its capacity to resist permanent deformation.

Figure 7b shows the fatigue factor (G*·sin δ) results obtained from the tests. It is found that the fatigue factor exhibits a trend similar to that of the rutting factor, with both decreasing as the temperature increases, and the virgin asphalt shows the lowest fatigue factor values. This can indicate that the virgin asphalt possesses superior fatigue resistance, whereas the addition of the RPP modifier slightly compromises the ability to resist fatigue damage, which slightly decreases in asphalt [42]. This behavior can be explained by the fact that RPP, regardless of whether it has been treated or not, behaves as a brittle material rather than an elastomer, and its elastic recovery capability is limited. Consequently, during the application and removal of cyclic loading, the incorporated modifier fails to effectively enhance, or may even reduce, the recovery deformation of the asphalt, meaning that the accumulated damage can more rapidly lead to fatigue cracking.

#### 3.1.3. Viscous-Elastic Transition Temperatures

**Figure 8:**
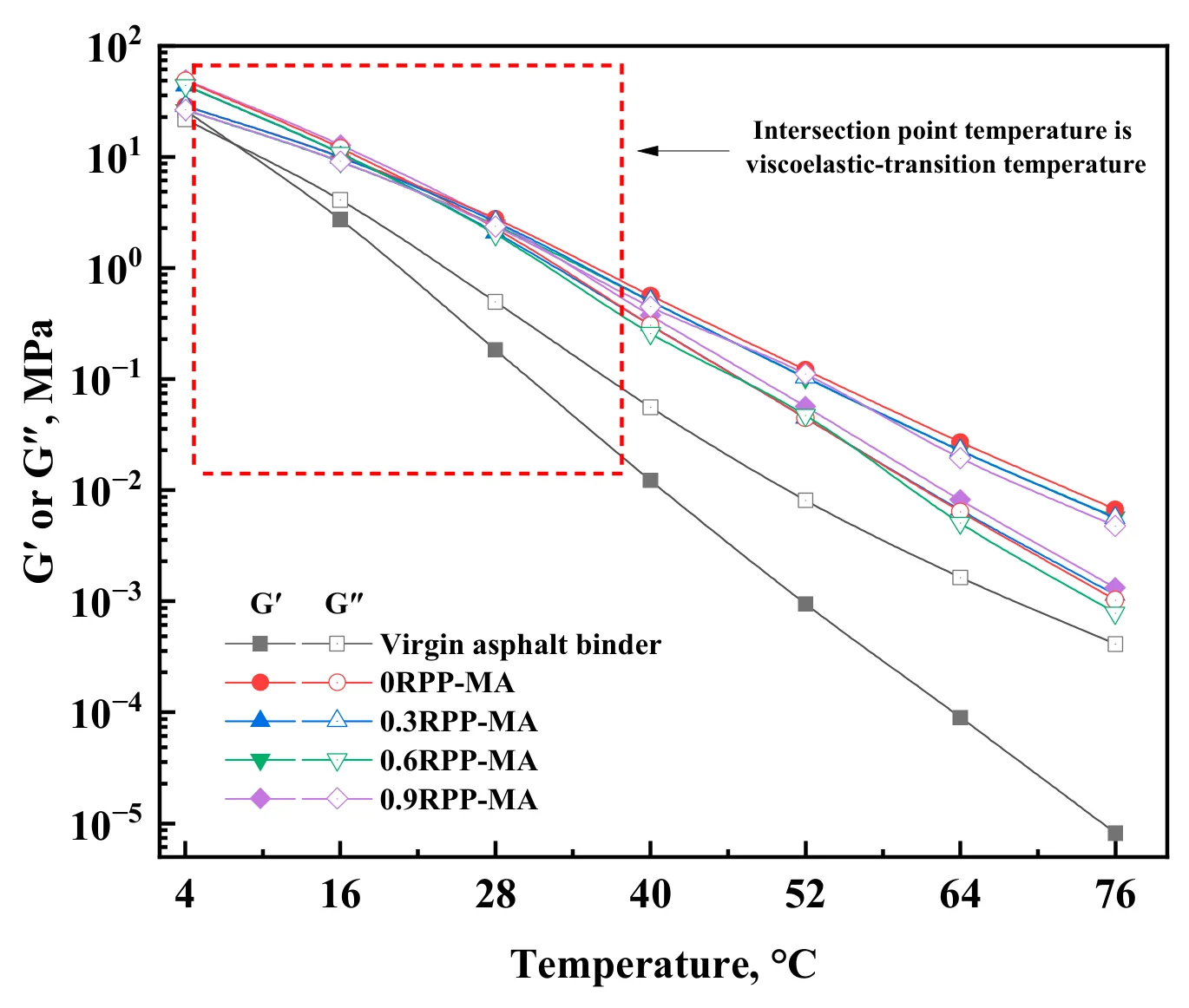
Viscous-elastic transition temperatures of RPP-modified asphalt at 10 rad/s.

Figure 8 presents the storage modulus (G′) and loss modulus (G″) of the different asphalt binders as a function of temperature. For each asphalt, it can be observed that G′ and G″ converge at a specific temperature, which is defined as the viscoelastic transition temperature, the point at which the rheological behavior of the asphalt shifts from elastic-dominant to viscous-dominant.

The viscoelastic transition temperatures of the various asphalts are listed in Table 4. As shown, the virgin asphalt exhibits the lowest transition temperature, at only 7.8 °C. Upon incorporation of the RPP modifier, the transition temperatures all exceed 20 °C, with the highest value, 25.7 °C, obtained for the 0.3RPP-MA formulation. These results can indicate that the addition of waste PP markedly shifts the rheological response of the asphalt from viscous-dominated to elastic-dominated, and among the tested modifiers, the formulation containing 0.3% DCP yields the lowest temperature susceptibility. This behavior can be attributed to the fact that waste PP is a non-polar crystalline plastic. Upon melting and blending into the asphalt, it forms a pronounced rigid skeleton structure, which effectively reduces the temperature susceptibility of the binder. Furthermore, compared with the case where no DCP is used for the chemical degradation of RPP, the addition of 0.3% DCP is able to disrupt the crystalline state of RPP and degrade it into lower-molecular-weight fragments. Such a treated modifier can be more readily molten and dispersed within the asphalt matrix, and the smaller molecular chains tend to distribute more uniformly and stably, which specifically manifests as enhanced elastic response and improved deformation resistance at elevated temperatures.

### 3.2. Frequency Response to Rheological Properties Under Various Environmental Temperatures

#### 3.2.1. Complex Modulus and Phase Angle

**Figure 9:**
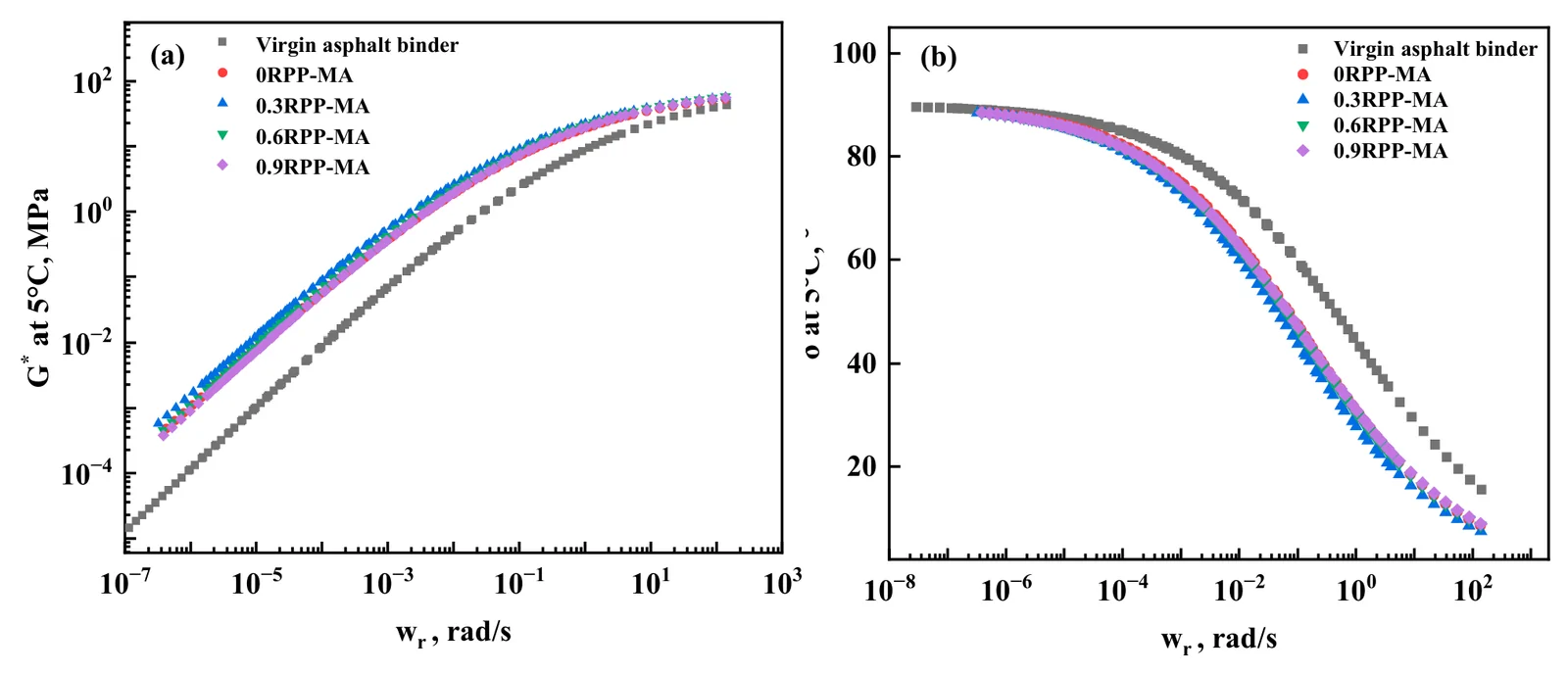
Master curves of complex modulus (**a**) and phase angle (**b**) at 5 °C for RPP-modified asphalt.

Figure 9 shows the complex shear modulus and phase angle of asphalt at 5 °C versus loading frequency. As frequency increases, G* rises while δ declines, indicating an elastic-dominant response under low-temperature conditions. The RPP-modified binders can exhibit significantly higher deformation resistance than the virgin asphalt at this temperature because asphalt, as a phase-change material, becomes stiffer and more brittle at low temperatures due to the reduced mobility of the oil component and hardening of the resinous fraction, leading to an essentially elastic mechanical behavior.

**Figure 10:**
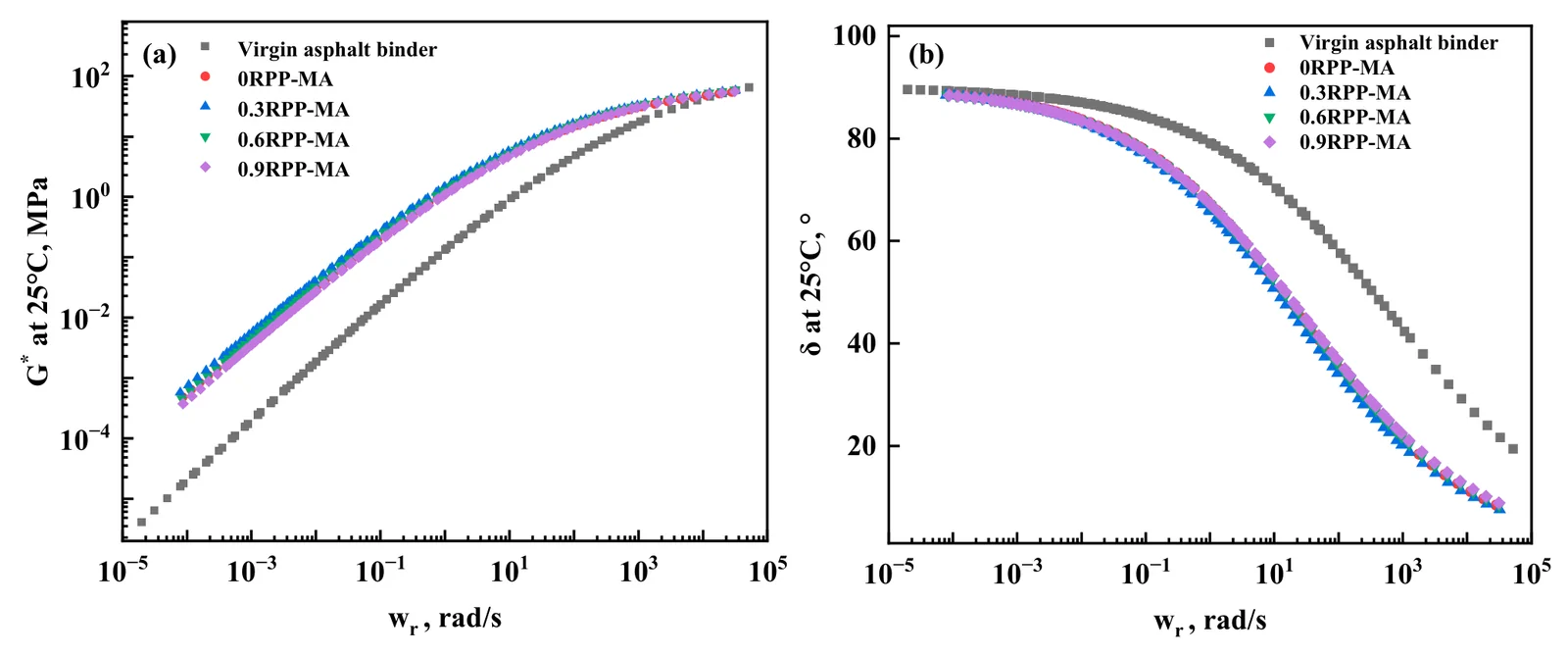
Master curves of complex modulus (**a**) and phase angle (**b**) at 25 °C for RPP-modified asphalt.

Figure 10 presents the complex shear modulus and phase angle of asphalt binders at 25 °C as a function of loading frequency. Similar to the trend at 5 °C, G* increases and δ decreases with increasing frequency, indicating that the binders still exhibit an elastic-dominant response and good deformation resistance under ambient conditions. Among the modified binders, that with 0.3% DCP shows the highest stiffness, while further increasing DCP content reduces stiffness. This is because an appropriate amount of DCP chemically degrades the long PP chains under heat and shear, producing lower-molecular-weight fragments that have a lower melting point and can form a more stable skeleton within the asphalt. However, excessive DCP induces crosslinking rather than further degradation. It is difficult for the crosslinked products to melt uniformly and they exhibit poor compatibility with asphalt, thus failing to enhance the deformation resistance.

**Figure 11:**
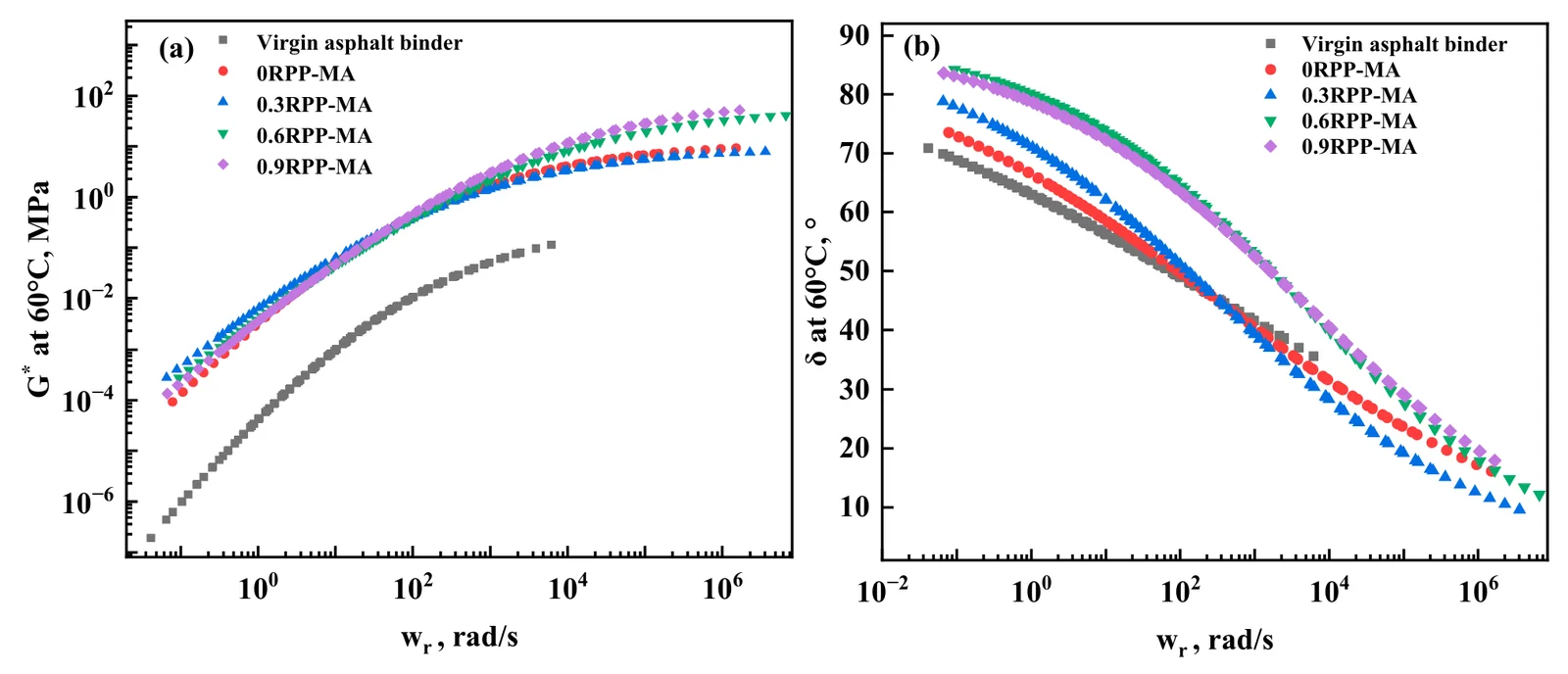
Master curves of complex modulus (**a**) and phase angle (**b**) at 60 °C for RPP-modified asphalt.

Figure 11 shows the G* and δ of asphalt binders at 60 °C versus loading frequency. The trends resemble those at low temperatures, but G* drops by two orders of magnitude. Under low-frequency loading, 0.3RPP-MA exhibits the highest G*, whereas at high frequencies, the asphalt modified with higher DCP content shows greater G. This indicates that although some elasticity remains at elevated temperatures, viscous behavior dominates. The variation among RPP-modified asphalt can be explained as follows: the modifier without crosslinking tends to lose elasticity under high-temperature, high-load conditions, leading to a viscous-dominant response. However, the crosslinked products generated by excess DCP have large molecular weights and high stiffness, thus still providing strong deformation resistance. This observation also confirms the occurrence of crosslinking reactions.

**Figure 12:**
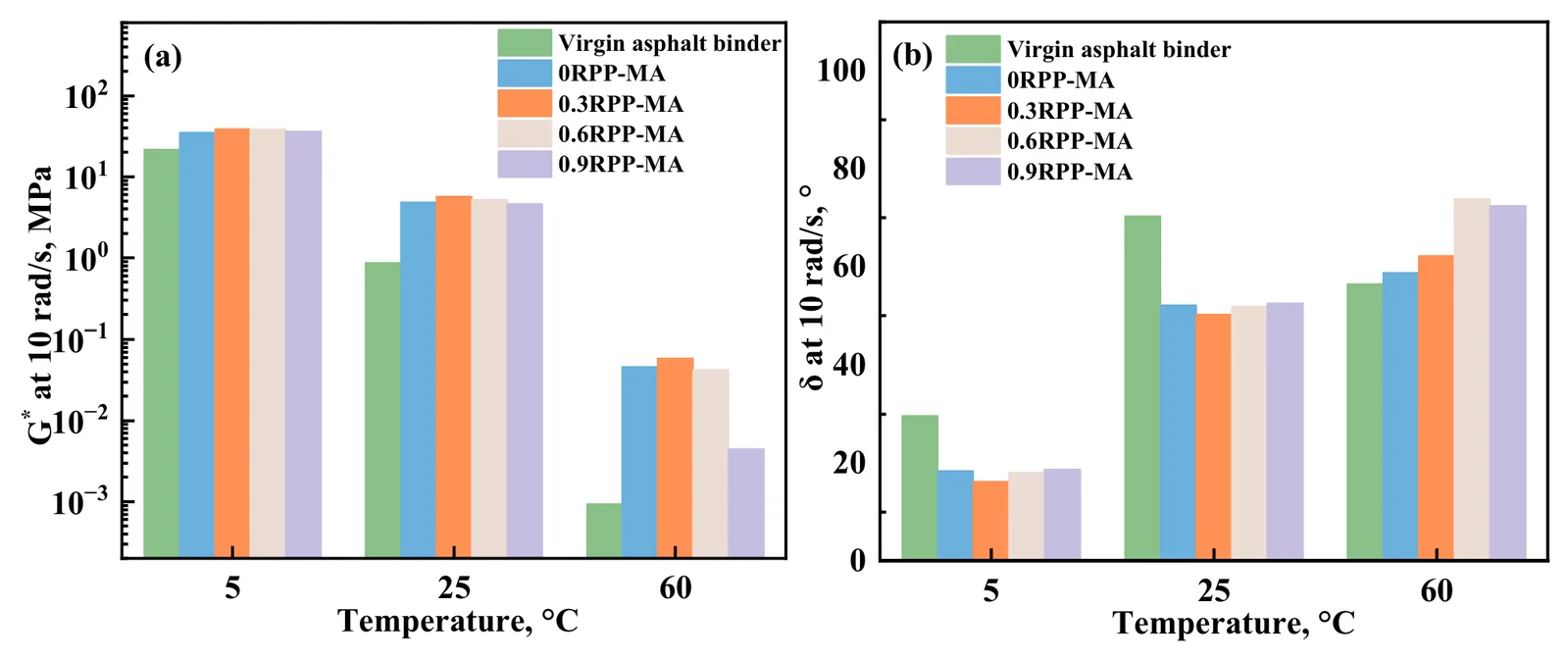
Complex modulus (**a**) and phase angle (**b**) at 10 rad/s for RPP-modified asphalt.

Figure 12 presents the complex shear modulus (a) and phase angle (b) of asphalt binders at various temperatures under a loading frequency of 10 rad/s. Overall, the rheological responses vary substantially with temperature. Specifically, at low temperatures, the G* values of different asphalts show little difference, whereas at elevated temperatures, 0.3RPP-MA exhibits the highest G*. Regarding the phase angle, all RPP-modified asphalts displayed significantly lower δ values than the virgin asphalt at low temperatures, with 0.3RPP-MA as the smallest. These results can indicate that the RPP modifier provides only marginal enhancement in deformation resistance at low temperatures, but markedly improves the elasticity and recovery capability at room temperature and above, with the 0.3RPP formulation being the most effective. The underlying reasons for this behavior have been discussed previously and are not reiterated here.

#### 3.2.2. Rutting Factor and Fatigue Factor

**Figure 13:**
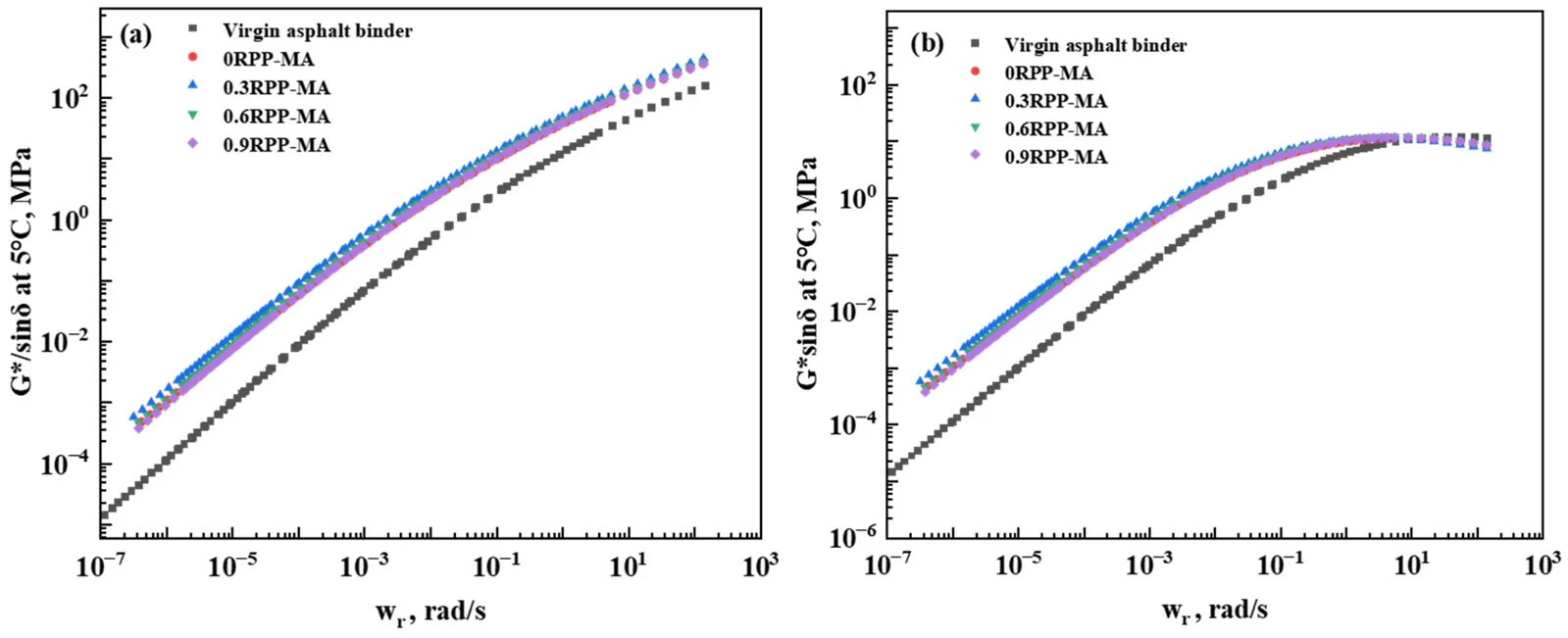
Master curves of rutting factor (**a**) and fatigue factor (**b**) at 5 °C for RPP-modified asphalt.

Figure 13 presents the rutting factor (a) and fatigue factor (b) of asphalt binders at 5 °C. At low loading frequencies, both factors increase with frequency, and the modified binders exhibit higher values than the virgin asphalt. At high frequencies, the fatigue factor decreases slightly, with the virgin asphalt showing the highest fatigue factor and the 0.3RPP-MA formulation presenting the most pronounced reduction. These results indicate that the RPP modifier significantly enhances the rutting resistance of asphalt at low temperatures, and also improves the fatigue resistance under high-frequency loading compared with the virgin asphalt. This is because the virgin asphalt, being fragile at this temperature, performs poorly under repeated loading, whereas the polymer network with adequate strength can better withstand fatigue damage. Moreover, when DCP is used for thermo-chemical degradation, the epoxy groups of ESO can ring-open and graft onto the PP chains, introducing flexible long chains that enhance the toughness of the modifier, which in turn contributes to the improved fatigue performance at high frequencies.

**Figure 14:**
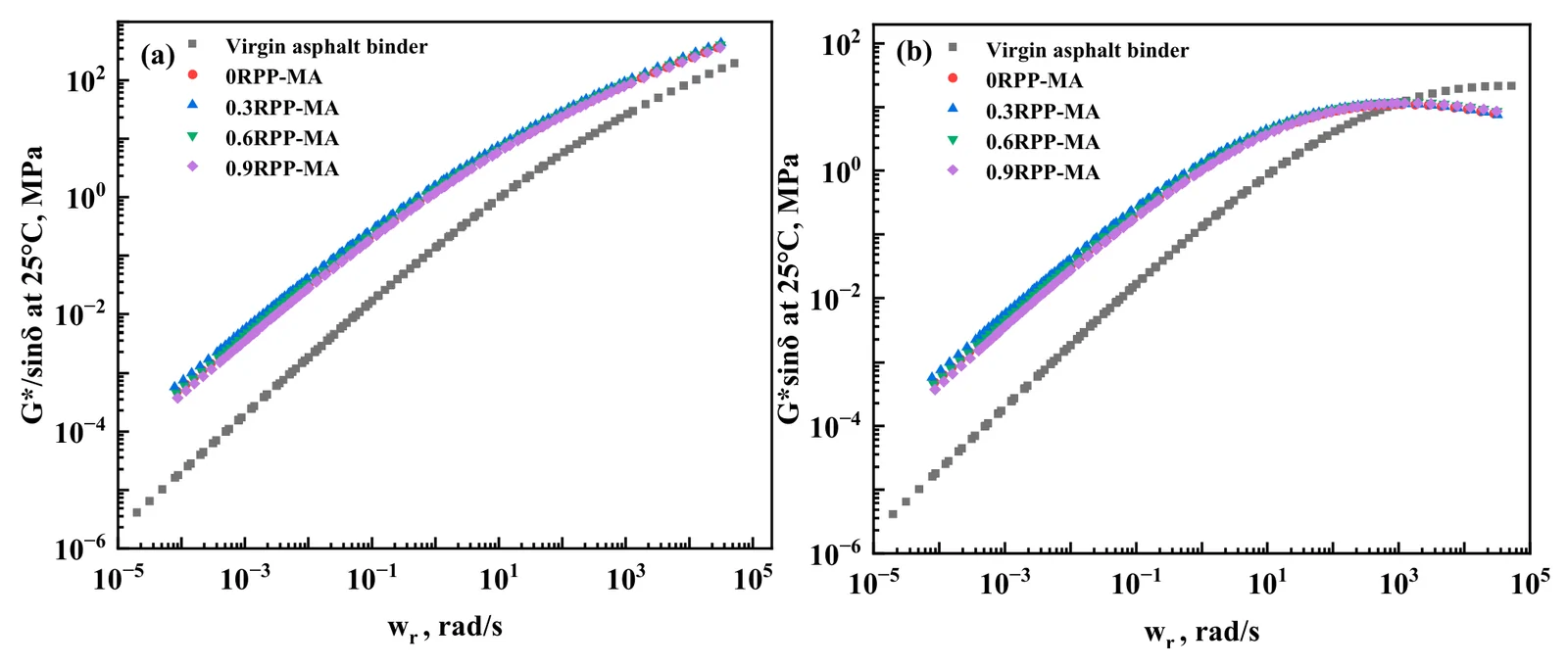
Master curves of rutting factor (**a**) and fatigue factor (**b**) at 25 °C for RPP-modified asphalt.

Figure 14 presents the rutting (a) and fatigue factors (b) of different asphalt binders at room temperature. The overall trends with frequency are similar to those at low temperature. However, at high frequencies, the fatigue factor of RPP-modified asphalts decreases markedly and falls well below that of the virgin asphalt. This can indicate that at ambient temperature, the viscoelastic response shifts from elastic- to more viscous-dominant, and the RPP-modified binders show significantly better fatigue resistance under high-frequency loading. The reason is that the virgin asphalt exhibits very poor deformation resistance under elevated temperature and high loading, whereas the polymer grafted with flexible ESO long chains gains enhanced toughness, and the skeleton structure formed by the RPP modifier provides strong resistance against repeated deformation under intense high-frequency loading.

**Figure 15:**
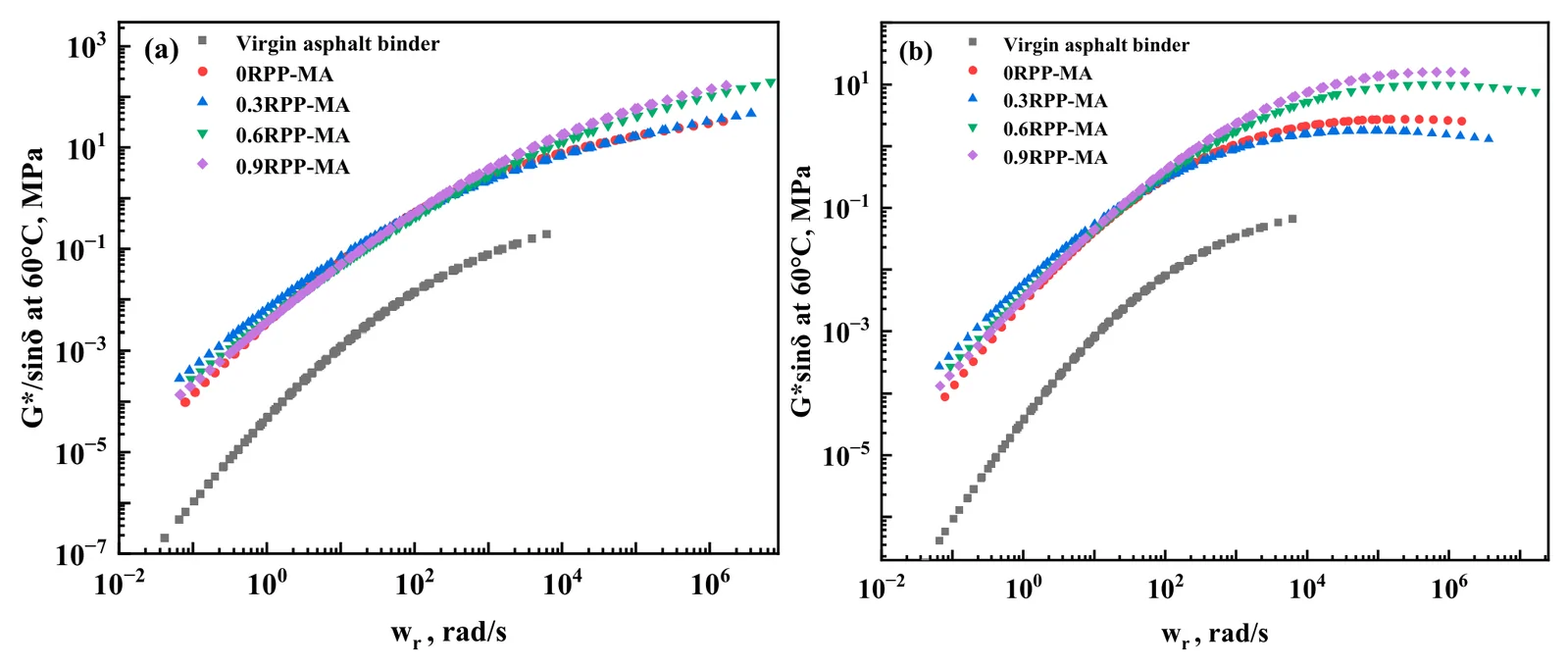
Master curves of rutting factor (**a**) and fatigue factor (**b**) at 60 °C for RPP-modified asphalt.

Figure 15 presents the rutting (a) and fatigue factors (b) of asphalt binders at 60 °C. The differences between RPP-modified and virgin asphalt are more pronounced than at low temperatures. For the rutting factor, 0.3RPP-MA exhibits the highest value at low frequencies, whereas 0.9RPP-MA becomes the highest at higher frequencies; both far exceed the virgin asphalt. For the fatigue factor, the virgin asphalt shows much lower values than the modified binders, with 0.3RPP-MA giving a relatively lower fatigue factor at high frequencies among the modified ones. These results can indicate that 0.3RPP-MA offers the best deformation resistance under typical road loading and demonstrates excellent durability under excessive loads. This is attributed to the rigid polymer structure that provides strong deformation resistance, while the flexible long chains grafted from ESO onto RPP enhance toughness, reduce brittle failure risk, and thus improve fatigue resistance and pavement durability.

### 3.3. FTIR Analysis

**Figure 16:**
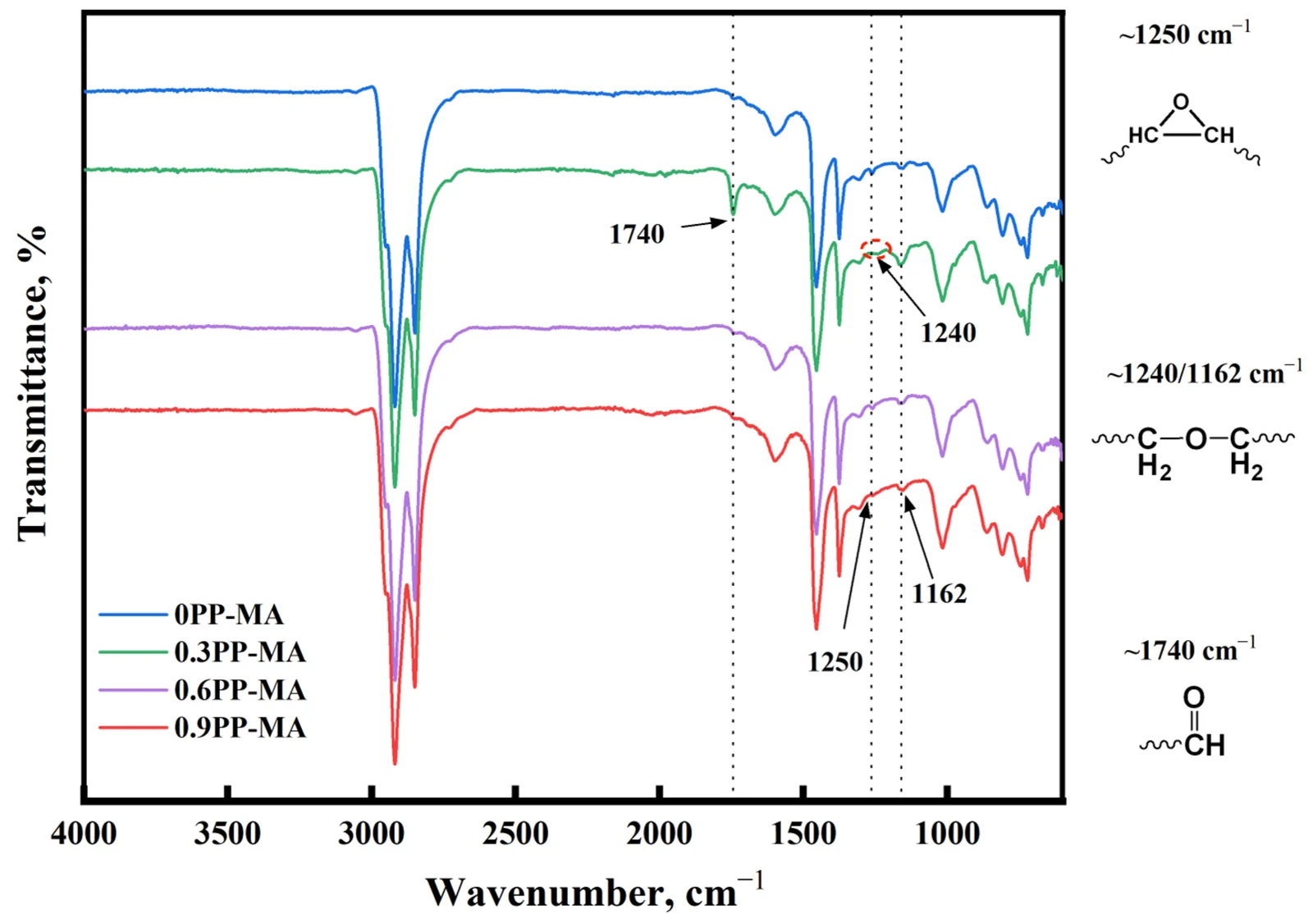
FTIR spectra testing for RPP-modified asphalt.

Figure 16 presents the FTIR spectra of the different RPP-modified asphalts, and the attributions of the main characteristic bands are listed in Table 5. Absorption bands at 3050 and 2725 cm^−1^ are assigned to hydroxyl (-OH) groups, which originate from oxidation of the waste RPP and partly from the asphaltene fraction of the virgin asphalt. For the modifiers without DCP or with 0.6% and 0.9% DCP, a stretching vibration peak of epoxide (CH(O)C) is observed at 1260 cm^−1^. However, this peak disappears when 0.3% DCP is used, and concurrently, more distinct ether (-C-O-C-) stretching peaks appear at 1162 and 1240 cm^−1^. This can indicate that, in the absence of DCP, ring-opening grafting of the epoxy groups in ESO cannot occur. With an appropriate DCP dosage, 0.3%, the thermally decomposed peroxide abstracts hydrogen from the PP chains, generating free radicals and inducing chain scission. It means that the epoxy groups have undergone a ring-opening reaction. It was proposed that this promotes radical attack on the epoxide groups, leading to ring-opening grafting, while the high-molecular-weight PP is degraded into lower-molecular-weight fragments. Compared with the 0%DCP system, the 0.3RPP-MA shows an additional aldehyde (-C=O) peak at 1740 cm^−1^, which is attributable to the fact that the limited number of grafting sites on the PP chains allows only partial ring-opened epoxide groups to be grafted, while the remainder are oxidized to aldehydes. In contrast, excessive DCP generates an overabundance of free radicals. Although the ring-opened ESO can be sufficiently grafted onto the radicals, the fragmented PP chains tend to undergo crosslinking, forming new extremely high-molecular-weight compounds that are not amenable to chemical degradation.

### 3.4. Mechanism Analysis

**Figure 17:**
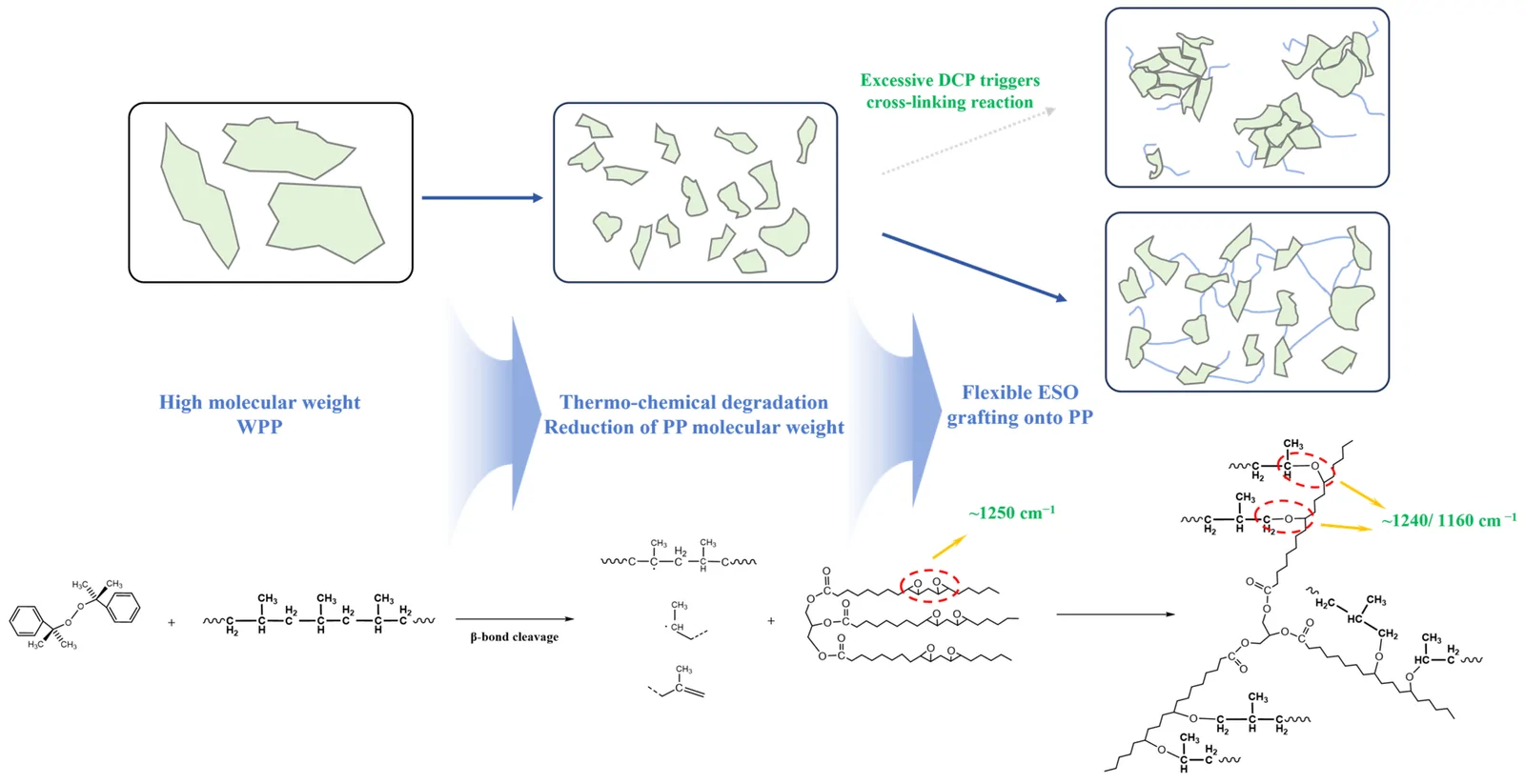
Mechanism analysis for RPP-modified asphalt.

Figure 17 shows that the chemical reactions involved in the RPP asphalt modifier can be divided into two main steps. First, a peroxide degradation agent is added to chemically degrade RPP at elevated temperatures, which lowers the melting temperature of the polymer and improves its dispersibility in asphalt [13]. When DCP is used for this treatment, an excessive amount induces crosslinking among the fragmented carbon chains, which not only prevents effective degradation but also generates more structurally complex network macromolecular products. Second, ESO undergoes ring-opening grafting reactions when Figure 17 shows that the chemical reactions involved in the RPP asphalt modifier can be divided into two main steps. First, a peroxide degradation agent is added to chemically degrade RPP at elevated temperatures, which lowers the melting temperature of the polymer and improves its dispersibility in asphalt [13]. When DCP is used for this treatment, an excessive amount induces crosslinking among the fragmented carbon chains, which not only prevents effective degradation but also generates more structurally complex network macromolecular products. Second, ESO undergoes ring-opening grafting reactions when the peroxide abstracts hydrogen from the polymer chains. According to the principle of similarity, the grafting of the oily ESO enhances the compatibility between the modifier and the asphalt matrix. Moreover, the introduction of flexible long chains from ESO increases the toughness of the RPP modifier, thereby improving the fatigue resistance of asphalt at low temperatures and compensating for the inherent brittleness and poor durability under repeated loading of polymeric materials. More importantly, ESO contains multiple epoxy groups, which provide multiple grafting sites after ring opening. The degraded low-molecular-weight PP can arguably thus be grafted to form a complex network structure. Within this structure, the rigid PP moieties contribute to deformation resistance under high-temperature and high loading conditions, while the flexible ESO long chains not only improve the dispersibility of the modifier but also enhance the fatigue performance of the asphalt [15].

## 4. Findings and Conclusions

This study proposes a method for preparing a modifier via the thermochemical degradation of RPP. For the modified asphalt produced with the RPP modifier, the optimal reagent ratio among the investigated formulations was determined through DSR experiments and CA model fitting, and the treatment mechanism and performance enhancement of the polymer modifier were elucidated in combination with FTIR analysis. The main findings are summarized as follows:

• The rheological properties based on temperature-sweep tests indicated that 0.3RPP-MA showed the highest viscoelastic transition temperature, 25.7 °C, compared with 7.8 °C for the virgin binder, showing better enhancement among the different formulations.
• The rheological properties based on frequency-sweep tests revealed that under varying pavement loading, the RPP-modified asphalts can exhibit superior fatigue resistance compared with the virgin asphalt. Particularly under heavy loading, the 0.3RPP modifier can also demonstrate satisfactory fatigue performance.
• Infrared spectroscopy (FTIR) results confirmed the radical induced ring-opening and grafting of ESO under thermochemical action and, from the perspective of reaction products, recommended 0.3% DCP as the suitable dosage in the modifier.
• Mechanism analysis of the recycling method demonstrates that the ring-open epoxy group can form a composite structured modifier with the degraded RPP, in which the rigid RPP and flexible ESO long chains together provide enhanced high-temperature performance and durability for the asphalt.

In summary, this study provides an efficient and green recycling route for RPP, and the 0.3RPP modifier is recommended for preparing RPP-modified asphalt. Although progress has been made in understanding the modification mechanism and performance of the modified asphalt, several issues still warrant further investigation, including its durability, aging resistance, low-temperature performance, and pavement performance under actual service conditions, as well as the life cycle assessment (LCA) of RPP-modified asphalt. In addition, systematic testing and evaluation of the linear viscoelastic region (LVE) are needed to further improve DSR rheological characterization. These aspects will be investigated in subsequent studies.

## Figures and Tables

**Table 1:** Physical properties of virgin asphalt.

Item	Test Result	Standard
Softening point (°C)	47.7	ASTM D36
Brookfield Rotational Viscosity, 135 °C (mPa·s)	463	ASTM D4402
Ductility, 25 °C (cm)	>100	ASTM D113
Penetration, 25 °C (0.1 cm)	65	ASTM D5

**Table 2:** Major information of DCP.

Item	Parameter
Molecular formula	C_18_H_22_O_2_
Purity, %	99
Melting point, °C	39~41
Number-average molecular weight	270.37
Density, g/cm^3^	1.56 (20 °C)

**Table 3:** Major information of ESO.

Item	Parameter
Molecular formula	(RC_2_H_2_OR′COO)_3_C_3_H_5_
Boiling point (°C)	150
Viscosity (cps, 25 °C)	325
Number-average molecular weight	1000
Density, g/cm^3^	0.990

**Table 4:** Viscous-elastic transition temperatures of RPP-modified asphalt.

Item	Viscous-Elastic Transition Temperatures/°C
Virgin asphalt binder	7.8
0RPP-MA	22.3
0.3RPP-MA	25.7
0.6RPP-MA	23.1
0.9RPP-MA	22.2

**Table 5:** Attribution of main characteristic bands of RPP-modified asphalt.

Wavenumber (cm^−1^)	Attribution	Vibration Type
3050, 2725	-OH	-OH stretching vibration
1740	-C=O	C=O stretching vibration
1600	-C=C-	Aromatic hydrocarbon C=C stretching vibration
1250	-CH(O)C-	Epoxy group CH(O)C stretching vibration
1240	-C-O-C-	Ethyl -C-O-C- stretching vibration
1162	-C-O-C-	Non-cyclic -C-O-C- stretching vibration

## Data Availability

The data presented in this study are available on request from the corresponding author.The data are not publicly available due to privacy or ethical restrictions.
